# Seven complete genome sequences of *Halomonas mongoliensis* isolates from hypersaline lakes in Brazilian Pantanal

**DOI:** 10.1128/mra.01043-25

**Published:** 2026-02-18

**Authors:** William Lautert-Dutra, Francine Melise dos Santos, Amanda Pasinato Napp, Clarissa Lovato Melo

**Affiliations:** 1 Environmental Monitoring and Biotechnology Laboratory, Institute of Petroleum and Natural Resources (IPR), Pontifical Catholic University of Rio Grande do Sul (PUCRS) 28102, Porto Alegre, Rio Grande do Sul, Brazil; California State University San Marcos, San Marcos, California, USA

**Keywords:** hybrid genome assembly, extremophile microorganisms, halophilic

## Abstract

Extremophilic microorganisms thrive in alkaline and saline conditions that reduce contamination risks, enabling efficient fermentation. We report the complete genomes of seven *Halomonas mongoliensis* isolates from salt-rich lakes in Brazil’s Pantanal, expanding resources for biotechnological development.

## ANNOUNCEMENT

The climate crisis has driven research into alternative solutions to mitigate climate change (1). Extremophilic microorganisms offer a biotechnological capacity for producing biomolecules and bioproducts (2). We studied seven halophilic strains to investigate their potential biotechnological capabilities related to the production of high-value molecules, including biosurfactants and other industrially relevant metabolites, and CO_2_ fixation through genomic data analysis.

Strains were isolated from sediment in a hypersaline lake in Brazilian Pantanal region (19°30′49″ S, 56°10′01.8″ W). The top layer (2 cm) of sediment was removed to eliminate contaminants. The apical portion (2 cm) of the remaining sediment was placed in 50 mL conical tubes and kept at 4°C. Isolation used saline medium (10 g/L yeast extract, 100 g/L NaCl, 3 g/L C_6_H_5_Na_3_O_7_, 2 g/L KCl, 1 g/L MgSO_4_, 280 μg/L MnCl_2_, 37 0.05 g/L FeSO_4_, and 3 g/L NaHCO_3_) (3, 4). Samples were added to tubes containing 3 mL of broth media (incubation at 28°C). Next, serial dilutions were performed using 0.9% saline solution, and aliquots were plated on saline medium agar (incubated at 28°C) until colonies were detected. Colonies were marked and cultured on solid saline medium to obtain morphologically homogeneous and pure isolates, which were then transferred to BHI medium, a rich and non-selective medium, for confirmation of purity. Each isolate was verified using Gram staining to discard contamination.

QIAcube equipment was used for DNA extraction (DNeasy Blood & Tissue, QIAGEN), and DNA quality control was performed using the Agilent TapeStation system (Agilent Technologies). Next, the DNA was sequenced using nanopore long-read technology (Oxford Nanopore Technologies). The sequence library was constructed using Native Barcoding Kit 96 V14 (SQK-NBD114.96) and enrichment of fragments > 3 kb (long fragment buffer[LFB] buffer). Library sequencing was done using FLO-PRO114M: PromethION R10 (M Version) at Life Sciences Core Facility (LaCTAD). Basecalling was performed using Oxford Nanopore’s Dorado basecaller (dna_r10.4.1_e8.2_400bps_sup@v4.3.0) super-accuracy model.

Long reads were processed using *Filtlong* v0.2.1 (“*minlength 1000 and keep percent 90*”) (5). Next, we used *Trycycler* v0.5.5 to generate consensus long-read assemblies for bacterial genomes (6). Multi-assembly used *Flye* v2.9.5-b1801, *Minipolish* (*miniasm_and_minipolish.sh*) v0.1.2, and *Raven* v1.8.3 (7–9). We manually curated assemblies using *Bandage* v0.8.1 and discarded fragmented genomes (10). The final consensus was polished using *Medaka* v2.0.1 (11, 12). Genome completeness was assessed using the *BUSCO* tool v5.8.2 (13). *QUAST* v5.2.0 tool was implemented to check genome assembly quality and coverage (14). Sequencing annotation was done using *PROKKA* v1.14.6 and *PGAP* v2024-07-18.build7555 (15, 16). We used *antiSMASH* v7 to annotate secondary metabolite biosynthesis gene clusters (17). The genomes were mapped to *Phylophlan* db v3.1.1 (18) using *DIAMOND* v2.1.11.165 (19). Multi-fasta files were aligned and trimmed using *MUSCLE* (v5.3.linux64) and *trimAL* v1.5 (20, 21). Gene trees were constructed with *RaxML v8.2.12* (ML + LG matrix-based model) (22). Final gene trees were generated using *TreeShrink* v1.3.9 (*-q “0.05, 0.10, 0.10”*) and *ASTRAL* v5.7.8 (23, 24). Genome assembly statistics and annotation features are detailed in Table 1.

**TABLE 1:** General statistics for genome assembly and annotation^*a*^

Features	BS215	BS221	BS222	BS224	BS226	BS238	BS256
SRA accession	SRX29202686	SRX29202685	SRX29202684	SRX29202683	SRX29202682	SRX29202681	SRX29202680
Raw reads count	294,368	153,627	275,233	239,304	656,639	322,714	203,485
Raw reads N50	14,181	15,752	15,657	19,355	13,735	16,827	11,027
Genome size (bp)	3,802,777	3,598,190	3,647,506	3,613,606	3,610,363	3,620,713	3,780,319
DNA G + C (%)	67.52	67.70	67.69	67.74	67.65	67.61	67.51
DNA scaffolds	1	1	1	1	1	1	1
N50	3,802,777	3,598,190	3,647,506	3,613,606	3,610,363	3,620,713	3,780,319
N90	3,802,777	3,598,190	3,647,506	3,613,606	3,610,363	3,620,713	3,780,319
L50	1	1	1	1	1	1	1
L90	1	1	1	1	1	1	1
N’s	0	0	0	0	0	0	0
Avg. coverage depth	381	250	404	454	669	636	246
Assembly accession	ASM5249606v1	ASM5249607v1	ASM5249635v1	ASM5249634v1	ASM5249656v1	ASM5249657v1	ASM5249676v1
BUSCO (halomonadaceae_odb12)	C: 97.4% [S: 97.1%, D: 0.3%], F: 0.5%, M: 2.1%, *n*: 973	C: 97.5% [S: 96.9%, D: 0.6%], F: 0.6%, M: 1.8%, *n*: 973	C: 97.6% [S: 96.9%, D: 0.7%], F: 0.6%, M:1.7%, *n*: 973	C: 97.6% [S: 96.9%, D: 0.7%], F: 0.6%, M: 1.7%, *n*: 973	C: 95.1% [S: 94.8%, D: 0.3%], F: 0.6%, M: 4.3%, *n*: 973	C: 97.3% [S: 96.7%, D: 0.6%], F: 0.6%, M: 2.1%, *n*: 973	C: 97.4% [S: 97.1%, D: 0.3%], F: 0.5%, M: 2.1%, *n*: 973
PGAP annotation - organism	*Halomonas mongoliensis* (ANI = 95.93%; Status: Inconclusive; Confidence: Low)	*H. mongoliensis* (ANI = 98.2%; Status: Confirmed; Confidence: High)	*Halomonas mongoliensis* (ANI = 98.42%; Status: Confirmed; Confidence: High)	*H. mongoliensis* (ANI = 98.42%; Status: Confirmed; Confidence: High)	*H. mongoliensis* (ANI = 95.9%; Status: Inconclusive; Confidence: Low)	*H. mongoliensis* (ANI = 95.93%; Status: Inconclusive; Confidence: Low)	*H. mongoliensis* (ANI = 95.95%; Status: Inconclusive; Confidence: Low)
Genes (total)	3,543	3,381	3,436	3,397	3,371	3,363	3,528
CDSs (total)	3,463	3,300	3,355	3,316	3,291	3,283	3,448
Genes (coding)	3,430	3,287	3,338	3,304	3,258	3,264	3,415
CDSs (with protein)	3,430	3,287	3,338	3,304	3,258	3,264	3,415
Genes (RNA)	80	81	81	81	80	80	80
rRNAs	4, 4, 4 (5S, 16S, 23S)	4, 4, 4 (5S, 16S, 23S)	4, 4, 4 (5S, 16S, 23S)	4, 4, 4 (5S, 16S, 23S)	4, 4, 4 (5S, 16S, 23S)	4, 4, 4 (5S, 16S, 23S)	4, 4, 4 (5S, 16S, 23S)
Complete rRNAs	4, 4, 4 (5S, 16S, 23S)	4, 4, 4 (5S, 16S, 23S)	4, 4, 4 (5S, 16S, 23S)	4, 4, 4 (5S, 16S, 23S)	4, 4, 4 (5S, 16S, 23S)	4, 4, 4 (5S, 16S, 23S)	4, 4, 4 (5S, 16S, 23S)
tRNAs	64	65	65	65	64	64	64
ncRNAs	4	4	4	4	4	4	4
Pseudo genes (total)	33	13	17	12	33	19	33
CDSs (without protein)	33	13	17	12	33	19	33
Pseudo genes (ambiguous residues)	0 of 33	0 of 13	0 of 17	0 of 12	0 of 33	0 of 19	0 of 33
Pseudo genes (frameshifted)	13 of 33	3 of 13	3 of 17	2 of 12	10 of 33	6 of 19	12 of 33
Pseudo genes (incomplete)	23 of 3	11 of 13	15 of 17	11 of 12	26 of 33	14 of 19	24 of 33
Pseudo genes (internal stop)	2 of 33	2 of 13	4 of 17	1 of 12	1 of 33	2 of 19	2 of 33
Pseudo genes (multiple problems)	4 of 33	3 of 13	4 of 17	2 of 12	4 of 33	3 of 19	4 of 33
CRISPR arrays	2	1	1	1	-	1	-
CDS	3,447	3,272	3,331	3,295	3,278	3,268	3,434
gene	3,545	3,369	3,421	3,385	3,374	3,359	3,530
mRNA	3,545	3,369	3,421	3,385	3,374	3,359	3,530
misc_RNA	19	16	10	10	16	11	17
rRNAs	12	12	12	12	12	12	12
repeat_region	2	2	2	2	-	1	-
tRNAs	66	68	67	67	67	67	66
tmRNA	1	1	1	1	1	1	1

^*a*^ The accession number for *Halomonas mongoliensis *is GCF_031451685.1.

**Fig 1:**
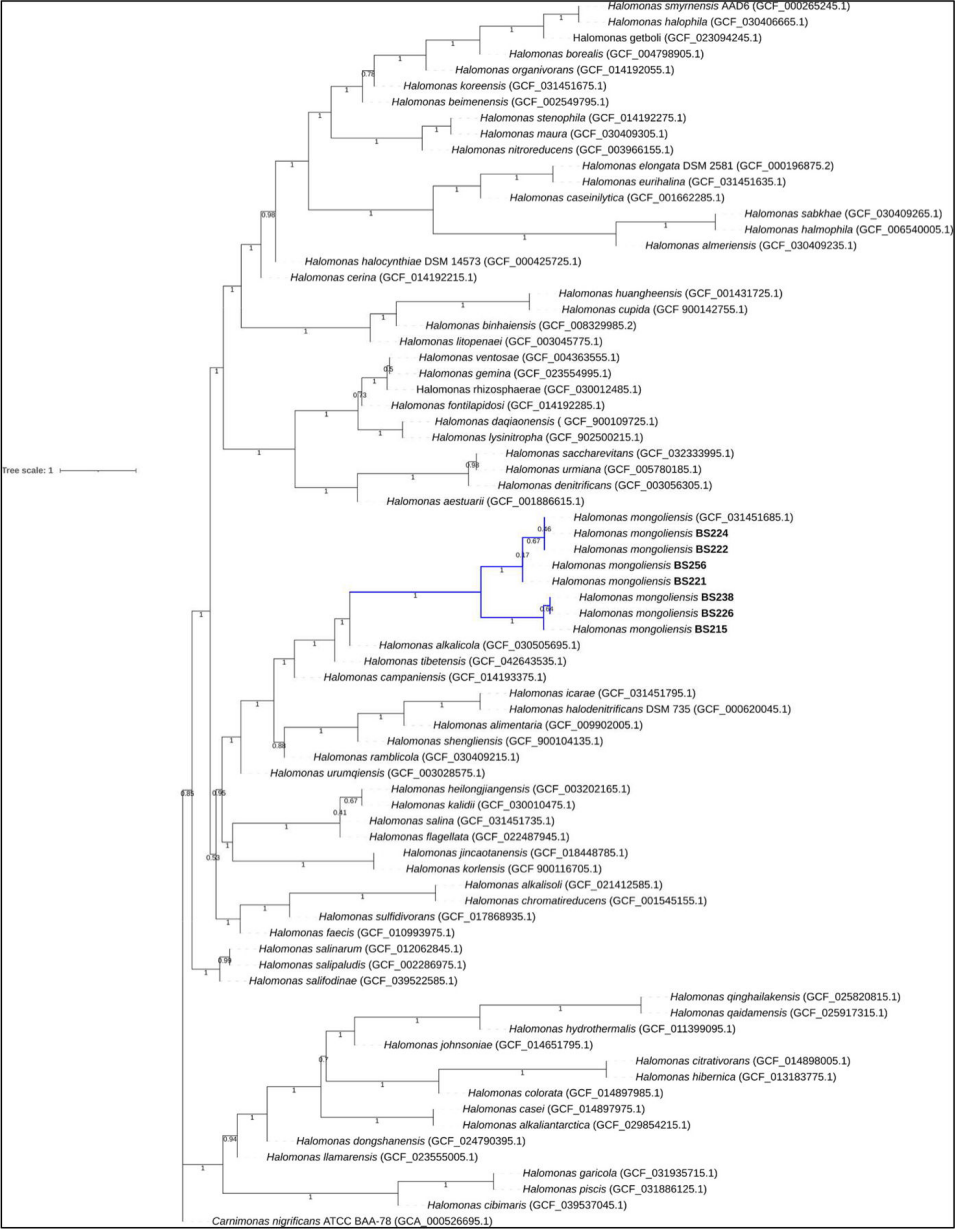
The phylogenetic species tree and correct placement of the *Halomonas mongoliensis* isolates. Sequence strains are highlighted in bold. Gene trees were inferred using *RaxML* (ML + G matrix-based model) for 400 genes in *Phylophlan* db. The final trees were trimmed using *TreeShrink* v1.3.9. The evolutionary species tree was generated using *ASTRAL*. The bootstrap value is shown next to the branches. The *Carnimonas nigrificans* genome was used as an outgroup.

The genomes of seven halophilic strains underscore the potential biotechnological capabilities of *H. mongoliensis*. Four isolates showed average nucleotide identity (ANI) values below 96% and formed a distinct subclade, underscoring intraspecies heterogeneity (Fig. 1). All genomes encode carbonic anhydrase genes, supporting their role in CO_₂_ fixation (25, 26). AntiSMASH revealed gene clusters for ectoine, Pf-5 pyoverdine, and lankacidin C, highlighting their metabolic value for sustainable, circular economy-driven applications (27).

## Data Availability

SRA sequences and assembly genomes are deposited under BioProject number PRJNA1274016. Additional files regarding genome annotation are deposited in 10.5281/zenodo.17298834.
